# Comparative Analysis of Outcomes in Acute Organophosphate Poisoning With and Without N-acetyl Cysteine Intervention

**DOI:** 10.7759/cureus.53155

**Published:** 2024-01-29

**Authors:** Muhammad Bilal Ahmed Attari, Tahir Zaman, Anza Amjad, Muhammad Haziq Khan, Zaineb Waqar, Saira Jabeen

**Affiliations:** 1 Internal Medicine, Lahore General Hospital, Lahore, PAK; 2 Emergency Medicine, Allied Hospital, Faisalabad, PAK; 3 Family Medicine, District Headquarter Hospital, Vehari, PAK; 4 Emergency Medicine, District Headquarter Hospital, Muzaffargarh, PAK; 5 Emergency Medicine, Mohi Ud Din Teaching Hospital, Mirpur, PAK

**Keywords:** icu mortality rate, mortality, pralidoxime, atropine, organophosphorus poisoning, n-acetyl cysteine

## Abstract

Introduction: Organophosphorus poisoning (OPP) stands as a significant health concern in numerous regions, especially in developing nations. Despite the rising complexities and case fatalities associated with exposure, the treatment approach has remained unchanged for many years. Based on clinical insights, certain pharmacologic agents have demonstrated utility in enhancing outcomes and reducing complications arising from this type of exposure.

Objectives: The objective of this study is to compare the outcome of N-acetyl cysteine in the treatment of acute organophosphate poisoning cases. In terms of a) its impact on the requirement of atropine, b) Length of hospital stay, and mortality.

Methods: The study was conducted in the intensive care unit (ICU) of the General Hospital Lahore. Thirty patients with a history and clinical presentation indicative of acute organophosphorus poisoning were randomly divided into two groups in a 1:1 ratio. The treatment group received parenteral administration of atropine, pralidoxime, and N-acetylcysteine (NAC) as an adjuvant, and the control group received standard treatment for acute organophosphate (OP) toxicity.

Result: Throughout the study duration, 30 patients suffering acute organophosphate (OP) toxicity (14 men, 16 women) were examined, with an age mean of (25.83±11.59) years. In the interventional group, only four patients required ICU admission, but in the control group, eight patients were admitted to ICU. The correlation result between the dose of atropine and length of hospital stays was not statistically significant between both study groups (<0.005). Plasma Cholinesterase (PChE) level (KU L−1) and total dose of Pralidoxime (g) were statistically significant in the length of hospital stay. The data was not normally distributed, so the non-parametric tests were applied. The Wilcoxon ranked test showed significant improvement in both the controlled and interventional groups because the p-value was (<0.005). Intergroup comparison analyzed by using the Mann-Whitney U test showed a significant reduction in the severity and other associated symptoms in the interventional group because the p-value was (0.001).

Conclusion: The outcome demonstrated that the NAC group had a decreased demand for atropine rather than Pralidoxime. In the NAC group, the length of hospital stay and mortality was decreased. The administration of NAC to the present study procedure for acute organophosphate (OP) poisoning is suggested.

## Introduction

Organophosphorus (OP) compounds are phosphorus-containing organic materials primarily used in pest control to replace environmentally persistent chlorinated hydrocarbons. Despite their amazing effectiveness against insects, these substances harm people [1]. Parathion, malathion, methyl parathion, dichlorvos, diazinon, phosmet, fenitrothion, tetrachlorvinphos, and azamethiphos are a few of the commonly used organophosphates. Malathion is the most commonly used organophosphate insecticide in the US. There are 40 approved organophosphate insecticides in the US, and they are used in both home and agricultural settings for a minimum of 73 million pounds annually [2].

In underdeveloped countries, OPs poisoning is a major source of both death and disease. It is responsible for about 200,000 fatalities and 3 million poisoning occurrences per year, which adds up to 5 million deaths during the previous years [3]. Even with this significant effect, research on potential therapeutics for this kind of toxicity is still scarce, and there are surprisingly few actively explored novel modalities [4]. Their main harmful mechanism is the suppression of acetylcholinesterase (AChE), which causes acetylcholine to build up in the body and acetylcholine receptors to be continuously stimulated [5]. Therefore, the objective of treating organophosphorus poisonousness is to inhibit cholinergic stimulation. This is accomplished by giving high dosages of atropine and using oximes as enzyme reactivators [6]. OP poisoning is associated with imbalances in other neurotransmitters, including catecholamines and Gamma-Amino Butyric Acid (GABA). Oxidative stress is mainly the pathogenic variable that has recently received much attention [7].

Glutathione (GSH) is a thiol-containing tripeptide that is widely distributed and is involved in several activities necessary for proper biological functions. These actions include the scavenging of free radicals and the detoxification of electrophilic xenobiotics. Organophosphates (OPs) induce oxidative stress, and some research has indicated that a medication such as N-acetyl-1-cysteine, which increases GSH content and acts as a reductant, may enhance the resistance to OP toxicity [8].

In a previous study, it was observed that administering N-acetyl-1-cysteine (NAC) to rats exposed to diazinon subchronically resulted in positive effects such as the reversal of oxidative stress indicators and the reactivation of acetylcholinesterase (AChE) [9] As a treatment for a variety of xenobiotic-induced poisoning wherein stress due to oxidation is suspected to play a role, NAC had come under greater attention over the past few years. NAC has not, however, been sufficiently investigated in humans for any of the aforementioned xenobiotic compounds to be categorically recommended as a medical approach. The increasing number of studies done recently has shown that stress due to oxidation may play a part in acute OP overdose, which leads to an understandable interest in NAC. This project aims to explore new treatment options for the treatment of OP poisoning and to lessen the death and disability associated with it. NAC is also used in various respiratory conditions, and respiratory failure is a common manifestation of OP poisoning. The objective of this study is to assess the outcome of N-acetyl cysteine in the treatment of acute organophosphate poisoning cases: a) In terms of its impact on the requirement of atropine, b) Length of hospital stay, mortality, and need for ventilation and ICU.

## Materials and methods

It was a Randomized Control Trial conducted in the Department of Medicine at Lahore General Hospital, a teaching hospital attached to Ameer-ud-Din Medical College in Pakistan. This title and research proposal was approved by the ethical review board committee of Post Graduate Medical Institute / Ameer-ud-Din Medical College/ Lahore General Hospital, Lahore, with registration no AMC/PGMI/LGH/ 00-177-20. This study was completed in 12 months duration. The sample size was calculated using WHO calculator version 12.2.6 using the reference study [10]. So the total sample size was n = 30 [10], and overall, 30 patients were divided into 15, 15 individuals in each group. A consecutive sampling technique was used in this study. 

Selection criteria

The selection criteria of the study were the following.

Inclusion Criteria

Individuals who have recently been acutely ill after being exposed to OP (male or female; 15 years of age or older; by any exposure method). Who has no background in diabetic mellitus, cardiac, pulmonary, renal, or hepatic disease? No hospital care for OP toxicity was provided in any hospital before the patient was admitted.

Exclusion Criteria

**Figure 1:**
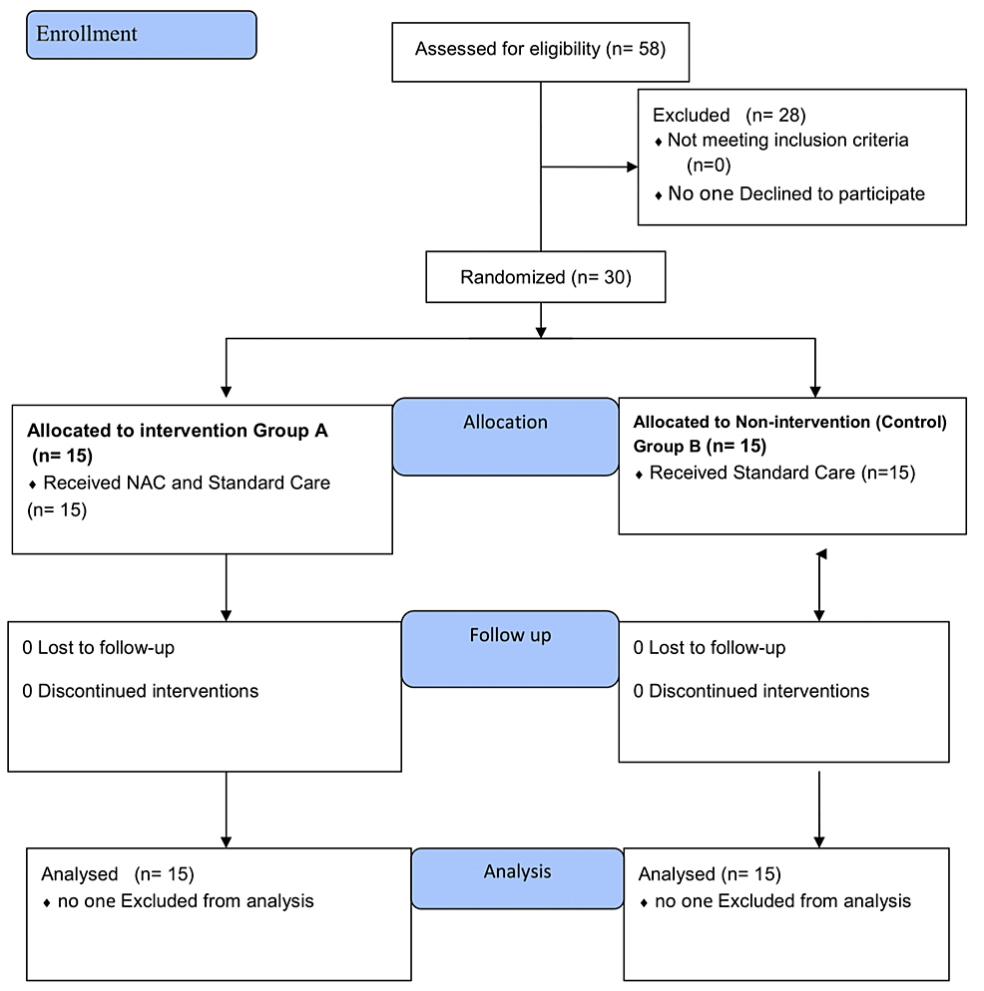
CONSORT Flow chart showing enrollment, intervention allocation, and follow-up of the patient

Women who are pregnant or breastfeeding. People who have ingested or been exposed to chemicals other than the OP. Individuals who have been exposed to the OP chemical more than 12 hours ago. A structured Performa was used to collect all the data after consent from the patients. In Figure 1, the CONSORT Flow chart shows enrollment, intervention allocation, and patient follow-up.

Ethical considerations

The guidelines and procedures set by the ethical board of Lahore General Hospital/Postgraduate Medical Institute, following the guidelines set by the University of Health Sciences, Lahore, were followed, and the rights of research participants were given due respect. Written informed consent (attached) was taken from all the participants. All information and data collection was kept confidential. Participants remained anonymous throughout the study. The participants were informed that the study procedure had no disadvantages or risks. They were also informed that they were free to withdraw at any time during the study process. Data was kept under key and locked while keys were kept in hand. On the laptop, it was kept under the password. Interventional Group: Individuals were selected at random to get NAC as an adjunctive therapy to atropine and Pralidoxime, along with receiving the standard therapy. N-acetylcysteine was given at the dose of 600mg three times daily for three days. This dose plan is known to reduce oxidative stress in various clinical studies. Standard protocols were atropine and pralidoxime. Control Group: Standard protocols were atropine and Pralidoxime. All participants received the standard care, which also included individual resuscitation, decontamination, and the injection of atropine and Pralidoxime in certain cases. Gastrointestinal lavage was performed on individuals who presented within two hours of OP consumption, and all individuals who had oral poison intake were given one dose of activated charcoal (50 mg). Any contaminated materials were thrown away, and detergent and water were used, if needed, to perform cutaneous decontamination. The data from both groups were collected.

Data analysis procedure

Written consent was obtained from attending family members. The history and clinical symptoms were used to diagnose organophosphorus poisoning. Miosis, increased secretions, diaphoresis, bronchospasm, and bradycardia, five typical clinical signs of OP poisoning, were chosen as parameters, and patients' severity was evaluated on a three-point scale ranging from 1-3. Salivation, lacrimation, urination, diarrhea, and vomiting are examples of increased secretions. A full history and examination were conducted on admission. These were also used to determine the degree of toxicity upon admittance and to confirm the acute exposure to OPs. Arterial specimens were gathered for thorough biochemical evaluations. The recommended care for severe OP poisoning primarily comprises intravenous delivery of atropine and Pralidoxime.

Randomization between groups was done by using the randomized table. A comparison was made between quantitative variables such as age, pulse, temperature, respiratory rate, length of hospital stay, and atropine requirement was done both pre-intervention and post-intervention and analyzed by the Wilcoxon sign ranked test. Qualitative variables such as gender, mortality, and need for ICU or ventilation were compared in both groups. Further data were gathered on demographics, place of residence, disease information, clinical evaluation information, treatment information, comorbid conditions, investigational information, adverse drug reactions, and treatment result information.

Outcome and utilization

Patients with acute organophosphate poisoning are increasing gradually in medical emergencies due to its easy availability in rural areas. Through this study, we were able to know the outcome of the use of N-acetylcysteine in sick people with severe organophosphate toxicity. It may open the door to new treatment strategies. Conclusions and recommendations drawn would help in better management of these patients. 

Outcome measure

Severity, poisoning symptoms, mortality, the entire dose of atropine provided, the length of hospitalization, and the requirement for mechanical breathing or ICU were all included as outcome measures.

Statistical analysis

SPSS (statistical software for social sciences) version 27 was used to analyze all the data. The means and standard deviation (SD) of age were calculated. The importance of variations in making comparisons among the various organizations was assessed using one of the tests listed below: To compare 2 pairs of related quantitative (non-parametric) values, use the Wilcoxon signed rank test. We used the Mann-Whitney U test to contrast two sets of mathematical (non-parametric) information. Many variables were correlated using the Spearman correlation coefficient test. P-values of 0.05 were used to determine statistical significance.

## Results

In this study, we evaluated 30 patients; the overall findings in Table 1 explained the demographics, clinical features, and outcomes of poisoning cases. The mean age of 25.83 years suggests a relatively young cohort. The main daily dose of atropine and total Pralidoxime provides crucial medication management information across all patients. The gender distribution indicates a slightly higher percentage of females. A notable aspect is the unanimous suicidal nature of poisonings, with no accidental cases reported. Severity scores classify a significant proportion of cases as severe, emphasizing the gravity of the situations encountered. Delving into the interventional group (IG) and control group (CG) specifics, the age distribution illustrates a subtle difference, with the IG being marginally younger at 24.86 years and the CG slightly older at 26.8 years. Variations in atropine dosage and pralidoxime administration between the IG and CG shed light on potential distinctions in the management strategies applied. Gender distribution nuances show a higher percentage of males in the IG, contrasting with a higher percentage of females in the CG. Examining the delay time before seeking medical attention, both groups predominantly presented within the first hour, with similar patterns. The need for ICU or ventilation support unveils a noteworthy difference, with a lower percentage in the IG (26.7%) compared to the CG (53.3%). Despite these variances, both groups demonstrated a 100% survival rate, underlining the effectiveness of the treatment approaches employed. The mode of poisoning being exclusively suicidal in both groups emphasizes uniformity like cases encountered. Severity scores mirror the overall distribution, with a comparable prevalence of moderate and severe cases in both the IG and CG. The hospital stay duration analysis provides granularity, showcasing diverse lengths of stay, with a higher proportion of three-day stays.

**Table 1:** Characteristics and outcomes of organophosphorus poisoning patients: A comparative analysis of interventional and control groups

Parameters		All Patient (n=30)	Interventional Group (n=15)	Control Group(n=15)
		Mean ± SD	Mean ± SD	Mean ± SD
Age	In year	25.83±11.59	24.86±11.18	26.8±12.3
Main daily dose of atropine (mg)	In Mg	7.56±1.67	7.0±1.0	8.13±2.03
Total dose of pralidoxime (g)	In Gram	5240.0±25.87	4613.33±2045.85	5866.6±2972.89
Demographic parameters and other variables		Frequency (Percentage)	Frequency (Percentage)	Frequency (Percentage)
Gender	Male	14 (46.7%)	8 (53.3%)	6(40%)
	Female	16 (53.3%)	7(46.7%)	9(60%
Delay time	Less than 1 Hour	20 (66.7%)	9(60%)	11(73.3%)
	1 to 2 hours	8 (26.7%)	5(33.3%)	3(20%)
	2 to 3 hours	1 (3.3%)	1 (6.7%)	0
	Greater than 3 hours	1 (3.3%)	0	1 (6.7%)
Need for ICU or Ventilation required	Yes	12 (40%)	4 (26.7%)	8 (53.3%)
	No	18 (60%)	11 (73.3%)	7 (46.7%)
Survived Non-survived	Yes	30 (100%)	15 (100%)	15 (100%)
	No	0	0	0
Mode of Poisoning	Suicidal (%)	30 (100%)	15 (100%)	15 (100%)
	Accidental n. (%)	0	0	0
Severity Score (1-3)	Mild	0	0	0
	Moderate	11 (36.7%)	5 (33.3%)	6 (40%)
	Severe	19 (63.3%)	10 (66.7%)	9 (60%)
Hospital stay duration (In days)	1.00	3 (10.0%)	0	3 (20%)
	2.00	2 (6.7%)	0	2 (13.3%)
	3.00	11 (36.7%)	6 (40.0%)	5 (33.3%)
	4.00	3 (10.0%)	2 (13.3%)	1 (6.7%)
	5.00	2 (6.7%)	2 (13.3%)	0
	6.00	1 (3.3%)	0	1 (6.7%)
	7.00	2 (6.7%)	0	2 (13.3%)
	8.00	3 (10.0%)	2 (13.3%)	1 (6.7%)
	16.00	2 (6.7%)	2 (13.3%)	0
	25.00	1 (3.3%)	1 (6.7%)	0

Table 2 explains the statistically significant difference between each group's pre and post-value comparisons. The Wilcoxon Sign Rank test revealed that by comparing the pre and post-values in the interventional group (NAC + Standard) with the control group (Standard therapy), the mean rank and p-value of each health parameter were determined. Regarding the "Post Severity Score - Pre Severity Score," both groups improved significantly (interventional: mean rank 8.00, p-value 0.001*; control: mean rank 5.00, p-value 0.004*). The interventional group had no positive ranks with a highly significant p-value of 0.001* in "Post Vomiting Symptom - Pre Vomiting Symptom," while the control group had a (p-value of 0.083). With p-values (interventional: 0.001*; control: 1.000), both groups improved in "Post Diarrhoea Condition - Pre Diarrhoea Condition". Similarly, "Post Sweating Condition - Pre Sweating Condition" and "Post Bronchospasm Symptom - Pre Bronchospasm Symptom" Post Abdominal Colic Condition - Pre Abdominal Colic Condition" "Post Bradycardia Symptom - Pre Bradycardia Symptom" "Post Coma Status - Pre Coma Status" revealed significant improvements in the interventional group. Although the control group only exhibited significant improvement in "Post Coma Status - Pre Coma Status" with p-values(0.05). Both exhibited significant improvements for the interventional group, but the control group had non-significant p-values, showing a significant difference in the effectiveness of the interventions between the groups.

**Table 2:** Wilcoxon Sign ranked result of Interventional group and Control group *- Significant values

Interventional Group ( NAC + Standard)		Mean Rank	p-value	Control group (Standard therapy)		Mean Rank	p-value
Post Severity Score - Pre Severity Score	Negative Ranks	8.00	0.001*	Post Severity Score - Pre Severity Score	Negative Ranks	5.00	0.004*
	Positive Ranks	0.00			Positive Ranks	0.00	
Post Vomiting Symptom - Pre Vomiting Symptom	Negative Ranks	0.00	0.001*	Post Vomiting Symptom - Pre Vomiting Symptom	Negative Ranks	0.00	0.083
	Positive Ranks	8.00			Positive Ranks	2.00	
Post-Diarrhoea Condition - Pre-Diarrhoea Condition	Negative Ranks	0.00	0.001*	Post-Diarrhoea Condition - Pre-Diarrhoea Condition	Negative Ranks	0.00	1.00
	Positive Ranks	8.00			Positive Ranks	0.00	
Post-Sweating Condition - Pre-Sweating Condition	Negative Ranks	0.00	0.001*	Post-Sweating Condition - Pre-Sweating Condition	Negative Ranks	0.00	0.83
	Positive Ranks	8.00			Positive Ranks	2.00	
Post Bronchospasm Symptom - Pre Bronchospasm Symptom	Negative Ranks	0.00	0.001*	Post Bronchospasm Symptom - Pre Bronchospasm Symptom	Negative Ranks	0.00	0.25
	Positive Ranks	8.00			Positive Ranks	3.00	
Post-Abdominal Colic Condition - Pre-Abdominal Colic Condition	Negative Ranks	0.00	0.001*	Post-Abdominal Colic Condition - Pre-Abdominal Colic Condition	Negative Ranks	0.00	0.08
	Positive Ranks	8.00			Positive Ranks	4.00	
Post-Bradycardia Symptom - Pre-Bradycardia Symptom	Negative Ranks	0.00	0.001*	Post-Bradycardia Symptom - Pre-Bradycardia Symptom	Negative Ranks	0.00	1.00
	Positive Ranks	8.00			Positive Ranks	0.00	
Post-Fasiculation Symptom - Pre-Fasiculation Symptom	Negative Ranks	0.00	0.001*	Post-Fasiculation Symptom - Pre-Fasiculation Symptom	Negative Ranks	5.00	0.140
	Positive Ranks	8.00			Positive Ranks	3.50	
Post Coma Status - Pre Coma status	Negative Ranks	0.00	0.001*	Post Coma Status - Pre Coma status	Negative Ranks	0.00	0.008*
	Positive Ranks	8.00			Positive Ranks	4.00	

Table 3 explains the Mann-Whitney test findings, comparing the pre-and post-values of the group comparison and outcomes between two groups (Group A: NCA Plus Standard Care and Group B: Standard Care) across numerous health metrics. Pre and post-severity score measurements, vomiting symptoms, diarrhea conditions, sweating circumstances, bronchospasm symptoms, abdominal colic conditions, bradycardia symptoms, fasciculation symptoms, and coma status are among the parameters. The mean rank and p-values of the Mann-Whitney test comparing Group A (NCA Plus Standard Care) and Group B (Standard Care) across multiple health parameters were studied. There was no significant difference between the groups for the "Pre Severity Score," with mean ranks of 16.00 for Group A and 15.00 for Group B (p-value 0.710). However, in the category of "Post Severity Score," Group A had a lower mean rank (11.50) than Group B (19.50), demonstrating a significant improvement in severity scores for Group A (p-value 0.001*). Regarding "Pre-Vomiting Symptom," there was no statistically significant difference between groups (p-value 1.000), with both having mean ranks of 15.50. In contrast, with a p-value of 0.000*, "Post Vomiting Symptom" showed significant improvement for Group A (mean rank 21.50) compared to Group B (mean rank 9.50). Similarly, there were no significant differences between the groups for "Pre Diarrhoea Condition" and "Pre Sweating Condition" (p-values 1.000), but for "Post Diarrhoea Condition" and "Post Sweating Condition," Group A showed substantial improvements (p-values 0.000*). The same pattern was seen for "Pre Bronchospasm Symptom" and "Pre Abdominal Colic Condition" (p-values 1.000), with significant improvements in Group A after intervention (p-values 0.000*). Additionally, there were no significant differences between groups for "Pre Bradycardia Symptom," "Pre Fasiculation Symptom," and "Pre Coma Status" (p-values 1.000). However, significant improvements were observed in Group A post-intervention for these parameters (p-values 0.000*, 0.000*, and 0.001*, respectively). In conclusion, the Mann-Whitney test reveals significant improvements in several health indices for Group A compared to Group B, showing the efficacy of the NCA plus Standard Care intervention.

**Table 3:** Comparison of Mann-Whitney test results for clinical parameters between NCA plus standard care and standard care groups in organophosphorus poisoning patients NAC: N-acetylcysteine *- Significant values

	Groups	N	Mean Rank	Sum of Ranks	P-value
Pre Severity Score	Group A (NCA Plus Standard Care)	15	16.00	240.00	0.710
	Group B (Standard Care)	15	15.00	225.00	
Post Severity Score	Group A (NCA Plus Standard Care)	15	11.50	172.50	0.001*
	Group B (Standard Care)	15	19.50	292.50	
Pre Vomiting Symptom	Group A (NCA Plus Standard Care)	15	15.50	232.50	1.000
	Group B (Standard Care)	15	15.50	232.50	
Post Vomiting Symptom	Group A (NCA Plus Standard Care)	15	21.50	322.50	0.000*
	Group B (Standard Care)	15	9.50	142.50	
Pre Diarrhoea Condition	Group A (NCA Plus Standard Care)	15	15.50	232.50	1.000
	Group B (Standard Care)	15	15.50	232.50	
Post Diarrhoea Condition	Group A (NCA Plus Standard Care)	15	23.00	345.00	0.000*
	Group B (Standard Care)	15	8.00	120.00	
Pre Sweating Condition	Group A (NCA Plus Standard Care)	15	15.50	232.50	1.000
	Group B (Standard Care)	15	15.50	232.50	
Post Sweating Condition	Group A (NCA Plus Standard Care)	15	21.50	322.50	0.000*
	Group B (Standard Care)	15	9.50	142.50	
Pre Bronchospasm Symptom	Group A (NCA Plus Standard Care)	15	15.50	232.50	1.000
	Group B (Standard Care)	15	15.50	232.50	
Post Bronchospasm Symptom	Group A (NCA Plus Standard Care)	15	20.50	307.50	0.000*
	Group B (Standard Care)	15	10.50	157.50	
Pre Abdominal Colic Condition	Group A (NCA Plus Standard Care)	15	15.50	232.50	1.000
	Group B (Standard Care)	15	15.50	232.50	
Post Abdominal Colic Condition	Group A (NCA Plus Standard Care)	15	19.50	292.50	0.001*
	Group B (Standard Care)	15	11.50	172.50	
Pre Bradycardia Symptom	Group A (NCA Plus Standard Care)	15	15.50	232.50	1.000
	Group B (Standard Care)	15	15.50	232.50	
Post Bradycardia Symptom	Group A (NCA Plus Standard Care)	15	23.00	345.00	0.000*
	Group B (Standard Care)	15	8.00	120.00	
Pre fasciculation Symptom	Group A (NCA Plus Standard Care)	15	15.50	232.50	1.000
	Group B (Standard Care)	15	15.50	232.50	
Post Fasciculation Symptom	Group A (NCA Plus Standard Care)	15	20.00	300.00	0.000*
	Group B (Standard Care)	15	11.00	165.00	
Pre Coma status	Group A (NCA Plus Standard Care)	15	15.50	232.50	1.000
	Group B (Standard Care)	15	15.50	232.50	
Post Coma Status	Group A (NCA Plus Standard Care)	15	19.50	292.50	0.001*
	Group B (Standard Care)	15	11.50	172.50	

Table 4 explains the results of Pearson correlation analyses examining the relationships between hospital stay duration and the mean daily dose of atropine (in mg) and the total dose of Pralidoxime (in mg) across the entire sample of 30 patients. For the correlation between hospital stay duration and the mean daily dose of atropine, the Pearson correlation coefficient is reported as -0.128. The associated p-value is .500, and with a two-tailed significance test, this result suggests no statistically significant correlation between these two variables. In other words, the mean daily dose of atropine does not appear to have a significant linear relationship with the duration of hospital stay. On the other hand, for the correlation between hospital stay duration and the total dose of Pralidoxime, the Pearson correlation coefficient is reported as .401. The associated p-value is .028, indicating statistical significance. Therefore, there is a statistically significant positive correlation between the total dose of Pralidoxime and the duration of hospital stay.

**Table 4:** Correlation analysis of hospital stay duration with mean daily dose of atropine and total dose of Pralidoxime in organophosphorus poisoning patients *- Significant values

Pearson Correlation		Hospital stay duration (In days)
Mean daily dose of Atropine (In mg)	Pearson Correlation	-0.128
	Sig. (2-tailed)	0.0500
	N	30
Total dose of Pralidoxime (In mg)	Pearson Correlation	0.401^*^
	Sig. (2-tailed)	0.028
	N	30

## Discussion

This research examined the properties of NAC as an additional treatment to atropine and Pralidoxime. The decrease in the mean daily atropine dosage and length of hospitalization showed that NAC is effective in treating acute OPS toxicity, according to the results. Our previous animal work, which showed the advantage of NAC in diazinon-induced poisoning, supports this conclusion.

Provided the high death rate and high cost of hospitalization in OP-intoxicated instances on only one side [11] and the cost-effectiveness of NAC on the other (12), it can be determined that including NAC in the existing therapeutic regimen for severe OP poisoning may help handle OP intoxication. A small sample size was a natural constraint of this investigation, so it is advised that a multicenter randomized medical experimental to determine the precise outcome of this unique medication in a bigger group of patients may instituted [12].

Thousands of deaths and cases of illness worldwide, mainly in underdeveloped nations, are attributed to organophosphorus poisoning. Even with the administration of conventional antidotes (atropine, oximes, and benzodiazepines), the case-fatality rate for insecticide poisoning is between 10 and 20%. There may be a requirement for ventilation and oxygen. Patients may need additional treatment modalities in supplementation to the specific antidote treatment of organophosphorus poisoning. Global fatalities from exposure to organophosphorus should decline as a result of improved medical therapy of the condition [5].

 In this research, acute organophosphorus poisoning patients were treated with N-acetyl cysteine in addition to the traditional antidote protocol. The treatment was contrasted with that of a similar group of people who received only the traditional treatment procedure without this extra medication. Cases were diagnosed based on their histories, clinical symptoms, and laboratory tests that revealed serum cholinesterase levels that were 50% or below the enzyme's normal range. The clinical and analytical results for the patients were documented throughout a 24-hour observation period [13].

These findings are consistent with those of Shadnia et al. [10], who used NAC in patients who had been poisoned with organophosphate and discovered that the NAC group needed less atropine but not Pralidoxime. The researchers attributed the results to NAC's involvement in reducing oxidative stress brought on by organophosphates. The length of hospitalization was also shorter in the NAC group, according to the research. The authors suggested incorporating NAC into the current treatment regimen for acute OP poisoning (Mitra et al., 2023) [14].

In mice given an acute high dosage of fenthion poisoning, Yurumez et al. tested NAC and discovered a definite improvement in survival ratio in the NAC-supplemented cohort. Researchers assessed MDA and GPx in all the mice and discovered a noticeable improvement in the NAC-supplemented set as well. The writers decided that NAC had curative and preventative effects on fenthion poisoning and advised more research studies to determine the precise mechanism of NAC's protective effects in organophosphate poisoning [15].

According to Abdollahi and Karami-Mohajeri, oxidative stress could play an important role in the mechanism behind the toxicity of organophosphate (OP) chemicals. Buckley et al. underlined the necessity for novel treatments like sodium bicarbonate for pesticide poisoning. They also thought that the community fitness and investigation viewpoints' responses to this poisoning were insufficient, given insecticide toxicity is a major cause of fatalities and deaths throughout the world) [16].

 Although Junova L et al. thought that blood alkalization could be helpful in curing acute organophosphorus poisoning, they advised that further research be done to describe the dose and regimen [17]. Additionally, Husain et al. thought sodium bicarbonate had therapeutic and preventative benefits for treating organophosphate poisoning and reducing its consequences, such as OPIDN (Organophosphorus-induced delayed neuropathy) [18].

These results are consistent with those of Eddleston and Chowdhury, who claimed that sodium bicarbonate and antioxidants are just two of the numerous pharmacologic agents that can significantly lessen organophosphate toxicity. The writers suggested conducting numerous clinical trials so that beneficial drugs could be approved for regular therapeutic usage in cases of organophosphate toxicity [19].

## Conclusions

Organophosphorus toxicity poses a persistent challenge with limited clinical advancements over the decades. This RCT concluded that generating reactive oxygen species by organophosphates triggers damaging molecular pathways, leading to cell death. Notably, N-acetylcysteine (NAC) has demonstrated effectiveness in improving patient outcomes and reducing hospital stays without adverse effects. The positive impact of NAC prompts consideration for its integration into management protocols.
